# Artificial Language Training Reveals the Neural Substrates Underlying Addressed and Assembled Phonologies

**DOI:** 10.1371/journal.pone.0093548

**Published:** 2014-03-27

**Authors:** Leilei Mei, Gui Xue, Zhong-Lin Lu, Qinghua He, Mingxia Zhang, Miao Wei, Feng Xue, Chuansheng Chen, Qi Dong

**Affiliations:** 1 Center for Studies of Psychological Application and School of Psychology, South China Normal University, Guangzhou, China; 2 Department of Psychology and Social Behavior, University of California Irvine, Irvine, California, United States of America; 3 State Key Laboratory of Cognitive Neuroscience and Learning & IDG/McGovern Institute for Brain Research, Beijing Normal University, Beijing, China; 4 Center for Cognitive and Behavioral Brain Imaging and Department of Psychology, Ohio State University, Columbus, Ohio, United States of America; 5 Department of Psychology, University of Southern California, Los Angeles, California, United States of America; University Of Cambridge, United Kingdom

## Abstract

Although behavioral and neuropsychological studies have suggested two distinct routes of phonological access, their neural substrates have not been clearly elucidated. Here, we designed an artificial language (based on Korean Hangul) that can be read either through addressed (i.e., whole word mapping) or assembled (i.e., grapheme-to-phoneme mapping) phonology. Two matched groups of native English-speaking participants were trained in one of the two conditions, one hour per day for eight days. Behavioral results showed that both groups correctly named more than 90% of the trained words after training. At the neural level, we found a clear dissociation of the neural pathways for addressed and assembled phonologies: There was greater involvement of the anterior cingulate cortex, posterior cingulate cortex, right orbital frontal cortex, angular gyrus and middle temporal gyrus for addressed phonology, but stronger activation in the left precentral gyrus/inferior frontal gyrus and supramarginal gyrus for assembled phonology. Furthermore, we found evidence supporting the strategy-shift hypothesis, which postulates that, with practice, reading strategy shifts from assembled to addressed phonology. Specifically, compared to untrained words, trained words in the assembled phonology group showed stronger activation in the addressed phonology network and less activation in the assembled phonology network. Our results provide clear brain-imaging evidence for the dual-route models of reading.

## Introduction

A key component of reading is phonological access, that is, the association of visual forms of words with their sounds. Many behavioral and neuropsychological studies have suggested two distinct routes of phonological access [1]–[4]. For the indirect (or assembled phonology) route, visual words are transformed into phonology through grapheme-to-phoneme correspondences (GPC). It is believed that readers of alphabetical languages mainly rely on assembled phonology, although there are further variations between shallow orthography (e.g., Italian) and deep alphabetical orthography (e.g., English). For the direct (or addressed phonology) route, phonological access either is mediated by semantics or relies on direct associations between the visual forms of words and their sounds. For logographic languages such as Chinese, phonological access mainly relies on addressed phonology. Within alphabetical languages (especially those with deep orthography such as English), it is also believed that high-frequency words and orthographically irregular words are accessed mainly through the addressed phonology route. In contrast, low-frequency regular words and pseudowords are accessed through the assembled phonology route [3].

Much neuroimaging research has investigated the neural bases of addressed and assembled phonologies by comparing brain activations associated with different reading materials: familiar words versus pseudowords [5]–[15], orthographically irregular words versus regular words [7], [10], [12], and logographic versus alphabetic writing systems [16]–[20]. For instance, it has been found that, compared to familiar words, pseudowords elicited greater activations in a number of language-related areas [6], [10]–[12], [21]–[26], such as the left inferior frontal gyrus and occipitotemporal cortex. Previous studies have also reported that, compared with regular words, irregular words elicited more activation in the left orbital frontal cortex [10], [12]. Based on a meta-analysis of 35 previous neuroimaging studies, Jobard et al. [27] proposed that assembled phonology mainly depends on the left supramarginal gyrus, posterior superior temporal gyrus, and dorsal inferior frontal gyrus, whereas addressed phonology mainly relies on the ventral part of the lateral temporal cortex and the inferior frontal gyrus.

In spite of these theoretical discussions and empirical investigations, a clear dissociation of the two phonological access pathways has not yet been established. Existing studies based on natural language materials have usually failed to reveal strong qualitative differences between the two pathways. For example, stronger activation in the left inferior frontal gyrus was consistently found when brain activities during pseudoword reading were subtracted by those during familiar word reading, suggesting this region's role in assembled phonology [10]–[12], [21], [23], [25]. However, the reverse subtraction (familiar words minus pseudowords) showed very little difference and thus failed to identify regions for addressed phonology [21], [23], [25], [28]. Still, the exact localization of the brain regions for the two pathways of phonological access is still under debate. For instance, the definition of a key region responsible for assembled phonology, the temporoparietal area, varied from the posterior superior temporal gyrus, angular gyrus, to supramarginal gyrus in different studies [27]. Some studies also failed to reveal any difference in the temporoparietal regions when participants read pseudowords vs. familiar words [10], [11], [24].

The inconsistent results across studies can be attributed to at least two causes. First, reading a natural language often involves both neural pathways, whose relative contributions depend on word familiarity (i.e., familiar words rely more on addressed phonology than do unfamiliar words) [3], [29]. It is therefore not clear which pathway is used by a given reader when reading a particular word whose familiarity may vary across readers. Second, contrasts of natural language materials are often confounded by factors such as task difficulty, visual form, phonology, and semantics. Specifically, the familiar word and pseudoword conditions differ in task difficulty and in whether they involve semantic processing. Similarly, the irregular and regular word conditions, especially for low-frequency words, often differ in task difficulty (i.e., leading to the regularity effect, with regular words named faster than irregular words) [30]–[32]. Finally, logographic and alphabetic scripts differ in visual form, phonology, and task difficulty.

**Figure 1:**
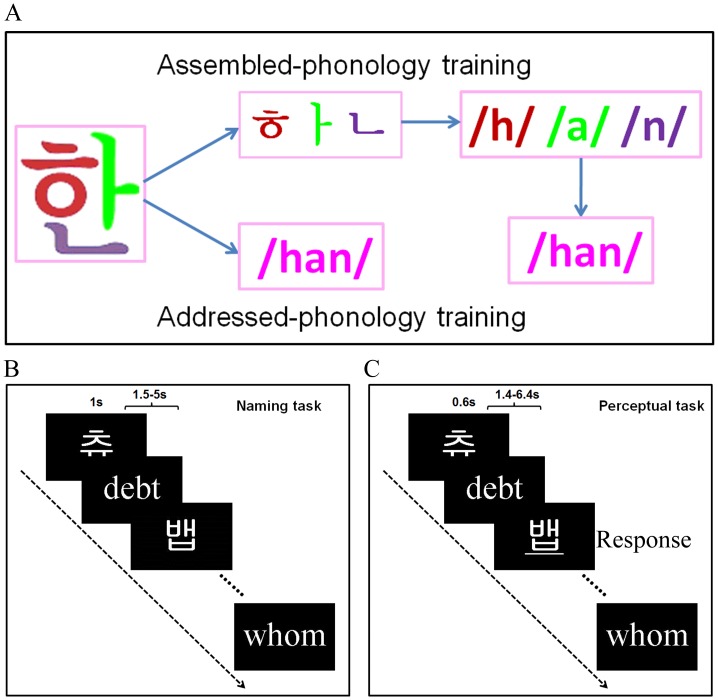
Experiment design and examples of the stimuli. Two matched groups of participants received addressed- and assembled-phonology training (A) for eight days (one hour per day). The naming task (B) was administered only after the training, while the perceptual task (i.e., underline detection, see part C) was administered both before and after the training.

To overcome these limitations and to clearly dissociate the neural pathways of addressed and assembled phonologies, we created an artificial language by adopting the visual forms and phonologies from 120 Korean Hangul characters (see Fig. 1A). Korean Hangul is ideal for our purpose because of its logographic visual appearance but alphabetic orthography [33]. In other words, Korean Hangul can read either through addressed or assembled phonology with the same visual forms. Semantics were excluded to avoid any potential effects of semantic processing on phonological access. The design of the artificial language in the addressed-phonology condition was the same as that used in Xue et al. [34] except that no semantics were provided. Therefore, this study investigated the addressed phonology that is not mediated by semantic. We trained two matched groups of native English-speaking participants in the U.S. with either addressed or assembled phonology, one hour per day for eight days. During fMRI scans, we used two reading tasks to investigate the neural mechanisms of addressed and assembled phonologies with different demand levels of phonological access: a naming task (Fig. 1B) that requires cognitive effort and a perceptual task (Fig. 1C) that emphasizes automatic phonological access.

Two specific issues were addressed in this study. First, we examined the neural mechanisms of addressed and assembled phonologies by comparing brain activation patterns elicited by trained words in the addressed group vs. the assembled group. It should be noted that the two groups were strictly matched on behavioral performance, visual complexity, and other linguistic factors such as visual form and phonology. The effect of semantics was also removed because the artificial words were not given meanings. Second, we examined the strategy-shift hypothesis, which postulates that practice can shift word reading strategy from sub-lexical (i.e., assembled phonology) to more automatic lexical reading (i.e., addressed phonology) [3], [29], [35]. We compared brain activation patterns elicited by trained words versus new words whose sounds had to be assembled based on their components in the assembled group. If the strategy-shift hypothesis were true, the trained words would elicit more activation in the addressed phonology pathway than would the novel words.

## Methods

### Participants

Forty-three native English speakers (20 males; mean age  = 21.19±1.97 years old, with a range from 19 to 27 years) participated in this study. They were divided into two groups: one was trained on “addressed phonology” (n = 21) and the other on “assembled phonology” (n = 22). The two groups were matched on nonverbal intelligence (Raven's Advanced Progressive Matrices) [36] and performance on English reading tasks [word identification and word attack from the Woodcock Reading Mastery Tests – Revised (WRMT-R) [37], phonemic decoding efficiency and sight word efficiency from the Test of Word Reading Efficiency (TOWRE)] [38] (Table 1). Based on participants' self-report, 9 participants in the addressed group and 8 participants in the assembled group were monolinguals. The remaining participants considered themselves as bilingual with their second language being one of the alphabetic languages (e.g., Spanish, French, or German). None of the participants had previous experience with Korean language. Because their second language was an alphabetic language, we believed that second language orthography would not be a significant confound when we contrasted the two groups. All participants had normal or corrected-to-normal vision, had no previous history of neurological or psychiatric disease, and were strongly right-handed as judged by Snyder and Harris's handedness inventory [39]. Informed written consent was obtained from the participants before the experiment. This study was approved by the IRBs of the University of California, Irvine and the University of Southern California.

**Table 1:** Participants' characteristics and their mean scores on reading tests and a nonverbal intelligence test.

	Addressed group	Assembled group	t	p
Handedness	90.11 (7.49)	89.42 (7.85)	0.30	.768
Age	20.67 (1.53)	21.68(2.23)	1.73	.091
Visual-auditory learning	122.29 (7.33)	123.64 (7.89)	0.58	.564
Word identification	97.76 (4.39)	98.73 (4.03)	0.75	.456
Word attack	38.24 (4.77)	38.36 (3.16)	0.10	.919
Sight word efficiency	98.92 (3.93)	99.45 (6.74)	0.31	.758
Phonemic decoding efficiency	55.42 (5.96)	56.80 (4.40)	0.86	.396
Raven's Advanced Progressive Matrices	24.95 (3.49)	26.23 (4.54)	1.03	.310

Note: Numbers inside the parentheses represent standard deviations. The handedness scores were calculated using the formula of [(Right – Left)/(Right + Left)] ×100, where a score of 100 represents complete right hander, while a score of −100 represents complete left hander. The scores for the reading and intelligence tests represent the number of correct items. The visual-auditory learning, English word identification, and English word attack are subtests of the Woodcock Reading Mastery Tests - Revised (WRMT-R); the sight word efficiency and phonemic decoding efficiency are subtests of the Test of Word Reading Efficiency (TOWRE).

### Materials

Sixty English words, 60 English pseudowords and 120 artificial language words were used in the study (see Fig. 1 for examples). All English materials were presented in gray-scale with 226×151 pixels in size, and the artificial language words were 151×151 pixels in size.

English words were selected from the MRC Psycholinguistic Database: Machine Usable Dictionary, Version 2.00 [40]. They were high-frequency monosyllable words (mean  = 530.80 per million, SD  = 740.98), 3–6 letters (mean  = 4.38, SD  = 0.85) in length. Monosyllabic English pseudowords were selected from the ARC Nonword Database [41], which matched the real words in number of letters (mean  = 4.38, SD  = 0.85).

The artificial language words were constructed using 22 Hangul letters (12 consonants and 10 vowels). We selected the phonemes that are easy to pronounce for native English speakers because this study focused specifically on learning form-sound association, not on learning new phonemes. To confirm our judgment, three native English speaking college students were asked to listen to the phonemes one-by-one and assess the ease of pronouncing the phonemes on a 5-point scale (1: very difficult to pronounce; 5: very easy to pronounce). The average scores across the judges were higher than 3 for each of the phonemes used in this study. The artificial language words were divided into two groups, one for training and the other (not trained) for examining transfer of learning. The two groups of words were strictly matched on the number of letters, as well as on the complexity and frequency of each letter.

The sounds of the English materials and artificial language materials (both words and phonemes) were recorded from a native English female speaker and a native Korean female speaker, respectively. All the sounds were denoised and normalized to the same length (600 ms) and loudness using Audacity 1.3 (audacity.sourceforge.net).

### Training Procedure

Using a computerized learning program, we trained participants to learn the association of visual forms and sounds of 60 artificial language words for eight days (one hour per day). Two training conditions (i.e., addressed-phonology and assembled-phonology training) were designed based on the same set of materials to contrast the neural bases of addressed and assembled phonologies (see Fig. 1A). In the addressed group, participants were asked to memorize each character as a whole. Because Korean Hangul has a shallow orthography with consistent correspondence between letters and their pronunciations in words/characters, participants would implicitly acquire the grapheme-phoneme correspondence (GPC) rules through learning if we used the original pronunciations of the letters. Thus, to avoid implicit acquisition of the GPC rules, we assigned each word with a new pronunciation (borrowed from one of the 60 artificial language words used for training in the study). In the assembled group, participants first learned the pronunciations of the 22 letters one by one and then assembled the phonology of the characters from their letters. In order to encourage the use of GPC rule instead of simply memorizing the association between letters or characters and their pronunciations, 30 new characters (untrained words consisting of learned letters) were tested at the end of each training session. For both groups, several types of learning tasks were designed to facilitate the acquisition of visual forms, sounds and their associations. They included naming, naming with feedback, fast naming (reading sets of ten words randomly selected from the 60 trained words as fast as possible), and a phonological choice task (selecting the correct pronunciation for the presented word from four sounds). It should be noted that, except for the type of training, all other variables such as time-on-task were controlled across the two groups.

### fMRI Task

Participants were scanned while performing two reading tasks (i.e., perceptual and naming tasks) often used in previous studies [10], [24], [34], [42], [43]. Both tasks consisted of four types of stimuli, namely English words, English pseudowords, trained artificial language words, and untrained artificial language words. Each type of materials contained 60 items. Stimulus presentation and response collection was programmed using Matlab (Mathworks) and the Psychtoolbox (www.psychtoolbox.org) on a laptop. Rapid event-related design was used for both tasks, with the five types of materials pseudo-randomly mixed. For both tasks, trial sequences were optimized with OPTSEQ (http://surfer.nmr.mgh.harvard.edu/optseq/) [44].

Two runs of the perceptual task were performed both before and after training (Fig. 1C). During each run, the stimuli were presented either in visual, auditory, or audiovisual modality. We focused on the visual modality in this paper because the purpose of this paper is to reveal the neural mechanisms of word reading. Each trial lasted for 600 ms, with a jittered inter-stimulus interval varying randomly from 1.4 to 6.4 sec (mean  = 1.9 sec) to improve the design efficiency. Participants were asked to carefully view and/or listen to the stimuli. To ensure that participants were awake and attentive, they were instructed to press a key whenever they noticed that the visual word was underlined. This happened 6 times per run. Participants correctly responded to 10.0±1.0 of 12 underlined words at the pre-training stage and 11.3±0.8 at the post-training stage, suggesting participants were attentive to the stimuli during the perceptual task.

The naming task also included two runs, which could only be performed after training (Fig. 1B). Each run consisted of 120 trials, with 30 trials for each condition. In each trial, a word was presented for 1000 ms, followed by a 1000 ms black interval. A jittered inter-stimulus interval varying randomly from 0.5 to 4 s (mean 1.2 s) was used to improve design efficiency. Participants were asked to read each visual word as fast and accurately as possible. Participants' responses were recorded through an MRI-compatible microphone connected to a laptop.

### Processing and Evaluation of Oral Responses

Participants' oral responses (reading out loud) recorded from the scanner were first denoised using Audacity 1.3 (http://audacity.sourceforge.net) to remove scanner noise. The reaction time (RT) for each trial was calculated using the following formula: RT  =  response time point (RTP) – trial onset. The RTP was defined as the first time point of 3 continuous points (within the time window of 300–2500 ms after the stimulus onset) whose intensity was higher than one standard deviation above the mean. The RTP was first automatically identified by a computer program on Matlab, and then manually checked one-by-one by the experimenter.

To calculate the accuracy of the naming task, we had two research assistants evaluate the sounds. The agreement rates between the two evaluators were very high: 97.59% (ranging from 91.67% to 100%) for English materials and 92.75% (ranging from 81.67% to 100%) for artificial words. Items that were initially scored differently by the two evaluators were evaluated by them jointly again to make final agreed-upon decisions.

### MRI Data Acquisition

Data were acquired with a 3.0 T Siemens MRI scanner in the Dana & David Dornsife Cognitive Neuroscience Imaging Center at the University of Southern California. A single-shot T2*-weighted gradient-echo EPI sequence was used for functional imaging acquisition with the following parameters: TR/TE/θ  = 2000 ms/25 ms/90^o^, FOV  = 192×192 mm, matrix  = 64×64, and slice thickness  = 3 mm. Forty-one contiguous axial slices parallel to the AC-PC line were obtained to cover the whole cerebrum and part of the cerebellum. An anatomical MRI was acquired using a T1-weighted, three-dimensional, gradient-echo pulse-sequence (MPRAGE) with TR/TE/θ  = 2530 ms/3.09 ms/10^o^, FOV  = 256×256 mm, matrix  = 256×256, and slice thickness  = 1 mm. Two hundred and eight sagittal slices were acquired to provide a high-resolution structural image of the whole brain.

### Image Preprocessing and Statistical Analysis

Initial analysis was carried out using tools from the FMRIB's software library (www.fmrib.ox.ac.uk/fsl) version 4.1.2. The first three volumes in each time series were automatically discarded by the scanner to allow for T1 equilibrium effects. The remaining images were then realigned to compensate for small head movements [45]. Translational movement parameters never exceeded 1 voxel in any direction for any participant or run. The images in the naming task were denoised using MELODIC independent components analysis within FSL [46]. An average of 11.60 components (ranging from 1 to 25) was removed from each scanning run. The images in the perceptual task were not denoised because there was little effect of head movements on the BOLD signal. All data were spatially smoothed using a 5-mm full-width-half-maximum Gaussian kernel. The smoothed data were then filtered in the temporal domain using a nonlinear high-pass filter with a 60-s cutoff. A 2-step registration procedure was used whereby EPI images were first registered to the MPRAGE structural image, and then into the standard (Montreal Neurological Institute [MNI]) space, using affine transformations with FLIRT [45] to the avg152 T1 MNI template.

At the first level, the data from the perceptual and naming tasks were separately modeled with the general linear model within the FILM module of FSL for each participant and each run. Events were modeled at the time of the stimulus presentation. The events' onsets and durations (600 ms for the perceptual task and 2000 ms for the naming task) were convolved with canonical hemodynamic response function (double-gamma) to generate the regressors used in the general linear model. Temporal derivatives and the 6 motion parameters were included as covariates of no interest to improve statistical sensitivity. Null events (i.e., fixation) were not explicitly modeled, and therefore constituted an implicit baseline. For the naming task, only correct responses were included in the analysis. The incorrect trials were modeled as nuisance variables to avoid their potential confounding effect. Eight contrast images (English words-baseline, English pseudowords-baseline, trained words-baseline, untrained words-baseline, English pseudowords-English words, trained words-English words, untrained words-English words, and trained words-untrained words) were computed separately by task and run for each participant.

Two second-level models (fixed-effects models) were separately constructed for the two reading tasks. For the perceptual task, training effect was calculated across the four runs (two at the pre-training stage and the other two at the post-training stage) for each condition and for each participant by using the contrast of post-training minus pre-training. For the naming task (administered after the training only), a second-level analysis was performed to average across the two runs for each participant.

For both tasks, the data from second-level analyses were then input into the third-level analyses which included three contrasts: 1) trained words-English words in the addressed group vs. trained words-English words in the assembled group; 2) trained words vs. untrained words in the assembled group; 3) English pseudowords vs. English words for all participants. One-sample and two-sample T tests were performed for within-subject and between-subject analyses, respectively. In these analyses, the two groups' English conditions were used as their own high-level baseline to control for potential group differences in baseline activation and test-retest variability of training effect. Group activations were computed using a random-effects model (treating participants as a random effect) with FLAME stage 1 only [47]–[49]. Unless otherwise indicated, group images were thresholded with a height threshold of z>2.3 and a cluster probability, P<0.05, corrected for whole-brain multiple comparisons using the Gaussian random field theory.

### Region of Interest Analysis

To examine whether the left precentral gyrus and fusiform gyrus are involved in the assembled phonology, we defined two regions of interest (ROIs) as two spheres each with a radius of 6 mm and centered at the peaks of activation found in the contrast of English pseudowords minus English words in the naming task (left precentral gyrus: x = −52, y = 0, z = 40; left fusiform gyrus: x = −46, y = −56, z = −20). The ROI analyses were performed by extracting parameter estimates (betas) of each event type from the fitted model and averaging across all voxels in the cluster for each participant. Percent signal changes were calculated using the following formula: [contrast image/(mean of run)] × ppheight ×100%, where ppheight is the peak height of the hemodynamic response versus the baseline level of activity [50].

## Results

### Behavioral Results

**Figure 2:**
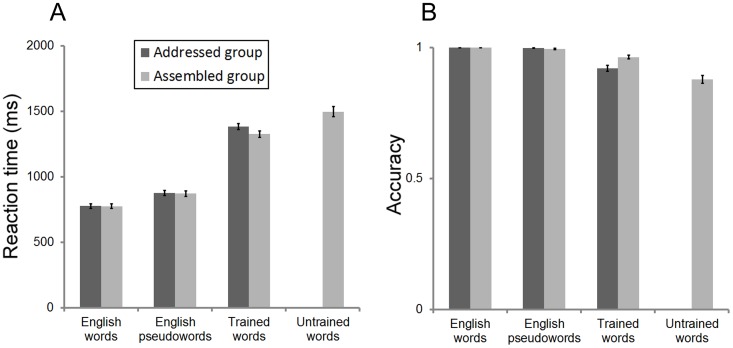
Reaction times (A) and accuracy rates (B) for the four types of words (i.e., English words, English pseudowords, trained artificial words, untrained artificial words) for the addressed and assembled groups. Error bars represent the standard error of the mean.

Behavioral results during the post-training scan showed that both groups correctly named more than 90% of the trained words (see Fig. 2). The assembled group also correctly named more than 85% of the untrained words. These results suggest that our training was effective and the assembled group had learned the GPC rules.

We then examined the behavioral differences between trained words in the addressed group and those in the assembled group, between trained words and untrained words in the assembled group, and between English words and pseudowords, because the subsequent analysis of fMRI data would focus on these three contrasts. First, we compared the trained words in the addressed versus assembled group by performing a two-way ANOVA [material (i.e., trained words and English words) and group (i.e., addressed and assembled groups)]. For reaction time (RT) the two groups did not show significant differences in the trained words (group: F(1,41)  = 1.52, n.s.; group-by-material interaction: F(1,41)  = 2.45, n.s.). For accuracy (CR), the assembled group performed better than the addressed group on the trained words, while there were no significant group differences for English words (group-by-material interaction: F(1,41)  = 10.89, p<.01). These results suggest that RT is matched between the trained words across the two groups. The differences in CR between the two conditions should not affect the subsequent fMRI analysis because we only included the correctly named words in the fMRI analysis.

Second, we compared the trained words and untrained words in the assembled group. Results showed that participants performed better on trained words than on untrained words, as indicted by the significant higher accuracy (t(21)  = 7.45, p<.001) and shorter RT (t(21)  = 5.26, p<.001). Potential effect of different RT on the BOLD response in this contrast was controlled statistically by adding RT as covariant in subsequent fMRI analysis.

Finally, we compared behavioral performance for English words versus pseudowords in the addressed and assembled groups. For both reaction time (RT) and accuracy rates (CR), participants performed better reading English words than pseudowords (RT: F(1,41)  = 349.76, p<.001; CR: F(1,41)  = 7.20, p = .01). Any potential effect of different RT on the BOLD response in this contrast was also controlled statistically by adding a covariate of demeaned RT in subsequent fMRI analysis. More importantly, the addressed group and assembled group did not show any significant differences for both RT (group: F(1,41)  = 0.01, n.s., group-by-material interaction: F(1,41)  = 0.10, n.s.) and CR (group: F(1,41)  = 1.65, n.s., group-by-material interaction: F(1,41)  = 2.46, n.s.), which further confirmed that the two groups were matched on English reading performance.

### Neural Bases of Addressed and Assembled Phonologies

**Figure 3:**
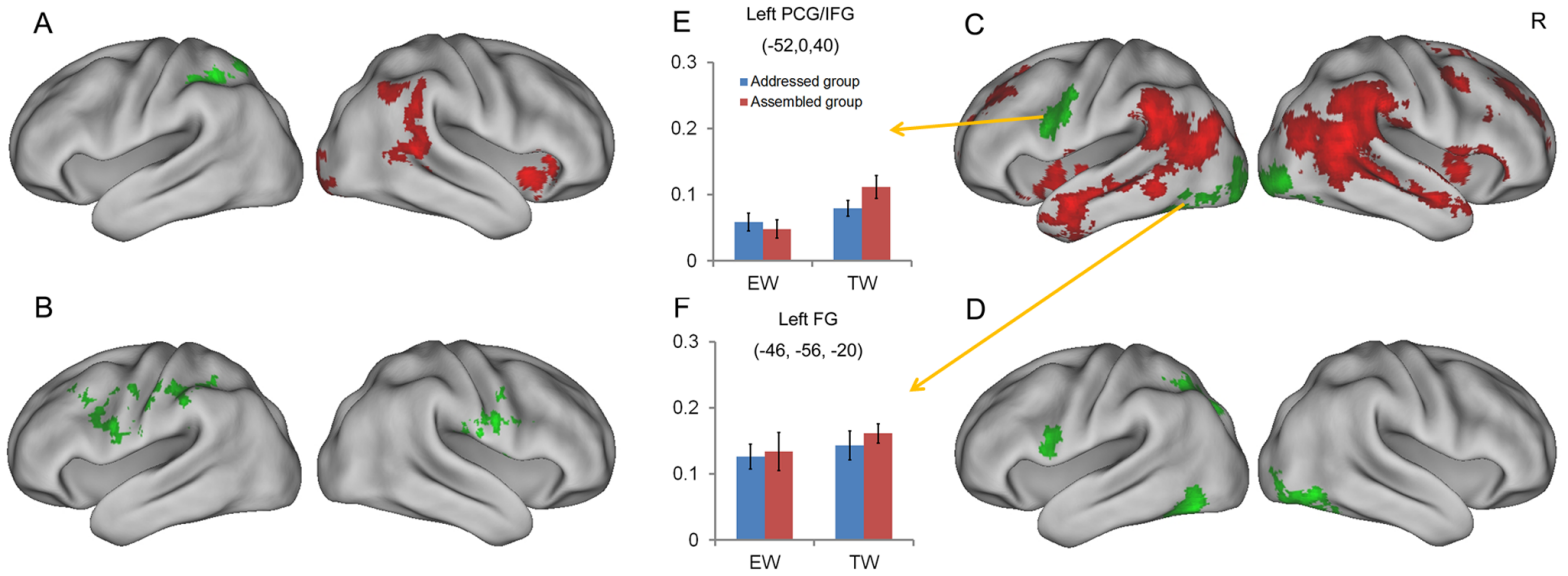
Neural pathways of addressed and assembled phonologies. Brain maps of trained words in the addressed group vs. those in the assembled group in the naming task (A) and in the perceptual task (B); brain maps of English words vs. English pseudowords in the naming task (C) and in the perceptual task (D). Red indicates regions showing more activations for the first element than the second in each contrast, while green indicates the reverse (second > first element). All activations were thresholded at z>2.3 (whole-brain corrected) and rendered onto PALS-B12 atlas [67], [68] via average fiducial mapping using caret software [69]. Bar graphs in the left panel were percent signal changes in two regions, i.e. the left precentral gyrus (PCG)/inferior frontal gyrus (IFG) and the left fusiform gyrus (FG), in the naming task. EW  =  English words; TW  =  trained words; and R =  right.

To reveal the neural bases of addressed and assembled phonologies, we first compared neural activities elicited by the trained words in the addressed group (relying on addressed phonology) with those elicited by the trained words in the assembled group (relying on assembled phonology) in the naming task. The BOLD responses in the English word condition were used as high-level baseline to control for group-related variability in BOLD response. As noted in the “Behavioral Results” section, behavioral performance on trained words in the naming task was matched in the two groups. Consequently, regions showing stronger activation for the addressed group's trained words were deemed as responsible for addressed phonology, whereas those showing stronger activation for the assembled group's trained words as responsible for assembled phonology. Results showed that greater activations for the assembled group were found in the left supramarginal gyrus [SMG, extending to superior occipital gyrus (SOG)] (see Tables 2 & 3), whereas greater activations for the addressed group were found in the right orbital frontal cortex (OFC) and middle temporal gyrus (MTG) (see Tables 4 & 5 and Fig. 3A). Anterior cingulate cortex (ACC), posterior cingulate cortex (PCC), and right angular gyrus (AG) also showed more activation in the addressed group.

**Table 2:** Brain regions showing stronger activation for assembled phonology than for addressed phonology.

Brain regions	EPW > EW				TW_AS > TW_AD				UW_AS > TW_AS			
	x	y	z	Z	x	y	z	Z	x	y	z	Z
*Naming task*												
Left PCG/IFG	−52	0	40	4.47					−54	12	22	4.46
Right PCG/IFG									56	8	26	3.87
Left SMG/SPL					−38	−38	40	3.07	−54	−36	50	3.23
Right SMG/SPL									36	−42	36	4.43
Left IOG/FG	−30	−96	−10	4.68								
Right IOG	34	−90	−10	4.93								
*Perceptual Task*												
Left PCG/IFG	−48	6	18	4.11	−56	6	24	3.73				
Right PCG/IFG					52	2	18	3.84				
Left SMG/SPL					−48	−34	42	4.25				
Left FG	−44	−54	−14	4.40								
Right IOG/FG	42	−70	−12	4.12								

Note: EPW  =  English pseudowords; EW  =  English words; TW_AD  =  the addressed group's trained words; TW_AS  =  the assembled group's trained words; and UW_AS  =  the assembled group's untrained words. PCG  =  precentral gyrus; IFG  =  inferior frontal gyurs; SMG  =  supramarginal gyrus; SPL  =  superior parietal lobule; ACC  =  anterior cingulate cortex; and FG  =  fusiform gyrus; IOG  =  inferior occipital gyrus.

**Table 3:** Cluster size (number of voxels) and cluster-level significance of brain regions for assembled phonology.

Brain regions	EPW > EW		TW_AS > TW_AD		UW_AS > TW_AS	
	Cluster size	p	Cluster size	p	Cluster size	p
*Naming task*						
Left PCG/IFG	958	<.001			2986	<.001
Right PCG/IFG					714	<.001
Left SMG/SPL			544	<.01	994	<.001
Right SMG/SPL					2964	<.001
Left IOG/FG	1460	<.001				
Right IOG	1335	<.001				
*Perceptual Task*						
Left PCG/IFG	408	<.05	694	<.001		
Right PCG/IFG			1010	<.001		
Left SMG/SPL			1030	<.001		
Left FG	1063	<.001				
Right IOG/FG	1081	<.001				

Note: EPW  =  English pseudowords; EW  =  English words; TW_AD  =  the addressed group's trained words; TW_AS  =  the assembled group's trained words; and UW_AS  =  the assembled group's untrained words. PCG  =  precentral gyrus; IFG  =  inferior frontal gyurs; SMG  =  supramarginal gyrus; SPL  =  superior parietal lobule; ACC  =  anterior cingulate cortex; and FG  =  fusiform gyrus; IOG  =  inferior occipital gyrus.

**Table 4:** Brain regions showing stronger activation for addressed phonology than for assembled phonology.

Brain regions	EW > EPW				TW_AD > TW_AS				TW_AS > UW_AS			
	x	y	z	Z	x	y	z	Z	x	y	z	Z
*Naming task*												
Right OFC/MFG	58	14	−2	5.01	36	18	−10	4.07				
ACC/left MFG	6	20	28	4.55	−6	34	12	4.04				
PCC	4	−38	44	4.90	6	−40	22	3.63	−4	−44	30	4.27
Left MTG/AG	−50	−8	−20	4.92					−62	−28	−14	3.46
Right MTG/AG	60	−52	12	5.85	50	−40	8	3.40				
Left AG									−52	−60	36	4.32
Right FG					30	−72	−16	3.54				
*Perceptual task*												
Left MTG/AG									−44	−56	14	3.70
Right MTG/AG									44	−46	12	3.26

Note: EPW  =  English pseudowords; EW  =  English words; TW_AD  =  the addressed group's trained words; TW_AS  =  the assembled group's trained words; and UW_AS  =  the assembled group's untrained words. OFC  =  orbital frontal cortex; MFG  =  middle frontal gyrus; ACC  =  anterior cingulate cortex; PCC  =  posterior cingulate cortex; MTG  =  middle temporal gyrus; AG  =  angular gyrus; and FG  =  fusiform gyrus.

**Table 5:** Cluster size (number of voxels) and cluster-level significance of brain regions for addressed phonology.

Brain regions	EW > EPW		TW_AD > TW_AS		TW_AS > UW_AS	
	Cluster size	p	Cluster size	p	Cluster size	p
*Naming task*						
Right OFC/MFG	1702	<.001	568	<.01		
ACC/Left MFG	8901	<.001	2226	<.001		
PCC	9998	<.001	760	<.001	638	<.001
Left MTG/AG	5755	<.001			296	<.05
Right MTG/AG	8357	<.001	1230	<.001		
Left AG					743	<.001
Right FG			658	<.001		
*Perceptual task*						
Left MTG/AG					704	<.001
Right MTG/AG					715	<.001

Note: EPW  =  English pseudowords; EW  =  English words; TW_AD  =  the addressed group's trained words; TW_AS  =  the assembled group's trained words; and UW_AS  =  the assembled group's untrained words. OFC  =  orbital frontal cortex; MFG  =  middle frontal gyrus; ACC  =  anterior cingulate cortex; PCC  =  posterior cingulate cortex; MTG  =  middle temporal gyrus; AG  =  angular gyrus; and FG  =  fusiform gyrus.

We then used the data from the perceptual task to compare the training effects for the trained words in both addressed and assembled groups. The English word condition was used as the baseline to control for test-retest fluctuations of the BOLD response. Specifically, the training effect was defined as follows: post-training contrast (i.e., trained words - English words) minus pre-training contrast (i.e., trained words - English words). Consistent with the results of the naming task, the left SMG showed greater activation for the assembled group than the addressed group (see Fig. 3B and Tables 2 & 3), but no regions showed more activation for the addressed group than the assembled group. The bilateral precentral gyrus [PCG, extending to the inferior frontal gyrus (IFG)] also showed more activation for the assembled group. Regions showing greater activation for addressed phonology than for assembled phonology in the naming task were not replicated in the perceptual task probably because of its lower demand on phonological access.

Finally, we compared the neural activities for English words versus pseudowords, i.e., the contrast that was often used by previous studies [10]–[12], [21], [23], [25], in both naming and perceptual tasks. We found English pseudowords elicited stronger activation in the left PCG/IFG, fusiform gyrus (FG), and bilateral inferior occipital gyrus (IOG) than English words (Table 2 and Fig. 3C&D). In contrast, English words showed stronger activation in an extensive network than pseudowords in the naming task, including ACC, PCC, right OFC, bilateral middle frontal gyrus (MFG), AG, and MTG (Tables 4 & 5 and Fig. 3C). However, no regions showed more activation for English words than pseudowords in the perceptual task (Fig. 3D).

In the above whole-brain analyses, the left PCG/IFG and FG showed more activation for English pseudowords than words. However, those two regions did not show greater activation for trained words in the assembled group than those in the addressed group in the naming task. To examine whether those two regions are involved in assembled phonology, we further extracted the percent signal changes from those two regions in the naming task. In the PCG/IFG, trained words in the assembled group elicited greater activation than those in the addressed group, but the activations for English words did not differ across the two groups (group-by-material interaction: F(1,41)  = 5.92, p<.05) (Fig. 3E), suggesting the left PCG/IFG is responsible for assembled phonology. In the left FG, the two groups did not show any differences for either English words or trained words (group-by-material interaction: F(1,41)  = 0.09, n.s.) (Fig. 3E), suggesting the left FG is involved in both addressed and assembled phonologies.

### Practice Shifts Word Reading Strategies from Assembled Pathway to Addressed Pathway

**Figure 4:**
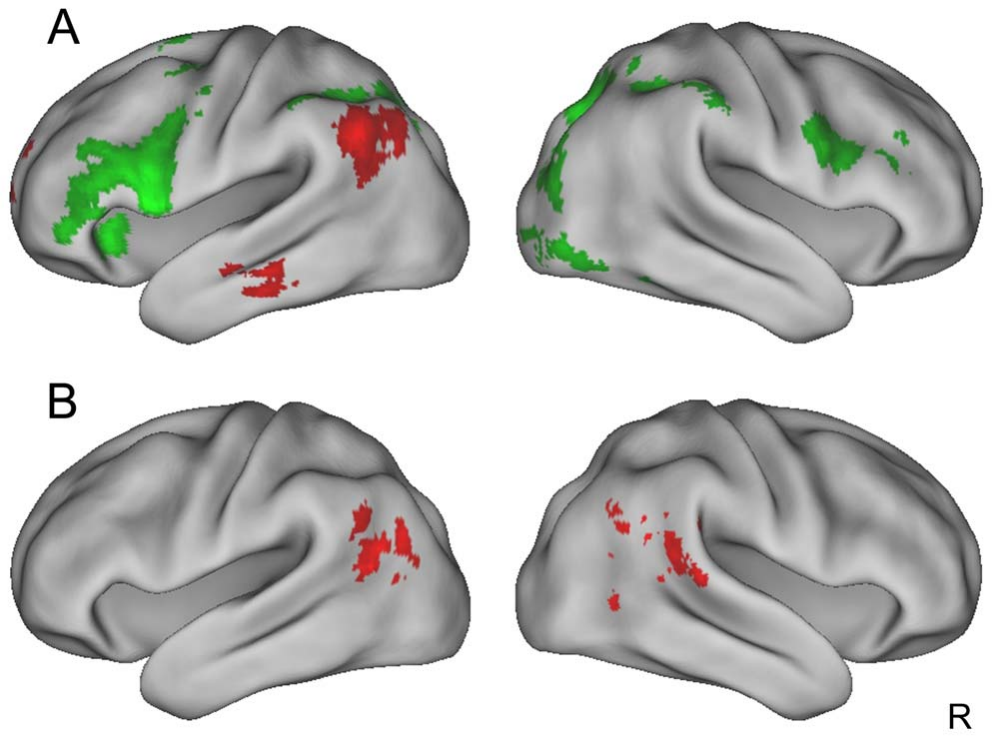
Brain regions showing differences between the assembled group's trained words and its untrained words in the naming task (A) and in the perceptual task (B). Red indicates regions showing more activations for trained words than untrained words, while green indicates the reverse (untrained words > trained words). All activations were thresholded at z>2.3 (whole-brain corrected) and rendered onto PALS-B12 atlas [67], [68] via average fiducial mapping using caret software [69]. R =  right.

Previous behavioral studies suggest that practice can shift participants' reading strategies from sub-lexical to more automatic lexical reading [3], [35]. We therefore hypothesized that the activation pattern for trained words in the assembled group should show more activation in regions for addressed phonology and less activation in regions for assembled phonology than untrained words in the assembled group. This hypothesis was confirmed: activation in the addressed phonology network (i.e., PCC, left MTG, and left AG) was stronger for trained words than for untrained words (Table 4 & 5 and Fig. 4A). In contrast, activation in the assembled phonology network (i.e., bilateral PCG/IFG and SMG) was weaker for trained words than for untrained words (Table 2). These results suggest that with increasing word familiarity, reading had a greater reliance on the addressed phonology pathway.

Similarly, in the perceptual task, training-induced increases in the addressed phonology network (i.e., bilateral MTG) for the trained words in the assembled group were greater than those for untrained words in the assembled group (see Fig. 4B and Tables 4 & 5). In contrast, training-induced increases in the assembled phonology network (i.e., the left PCG/IFG and SMG) were greater for the untrained words than the trained words in the assembled group, although they did not survive the stringent whole-brain correction perhaps due to the lower phonological demands of the perceptual task.

## Discussion

Using an artificial language training paradigm, we found clear evidence for separate neural substrates underlying addressed and assembled phonologies: (1) addressed phonology depended more on the ventral pathway, including the anterior cingulate cortex, posterior cingulate cortex, right orbital frontal cortex, angular gyrus and middle temporal gyrus, whereas assembled phonology relied more on the dorsal pathway, including the left precentral gyrus/inferior frontal gyrus and supramarginal gyrus; (2) training of addressed and assembled phonology increased activations in the respective pathways; and (3) the recruitment of the two neural pathways was modulated by word familiarity—familiar words recruited more of the addressed pathway and less of the assembled pathway than did novel words.

Compared with previous studies that relied on the contrasts of natural language materials [7], [10]–[12], [19]–[22], the artificial language training paradigm used in this study has several advantages: (1) words for the addressed and assembled groups were constructed using the same set of words, and consequently strictly matched on visual form and phonology; (2) the two groups were trained using the same procedure, matched on the number of repetitions and overall learning time; (3) semantic effects were removed because the artificial words were not given meanings; and (4) the contrast between addressed phonology (i.e., trained words in the addressed group) and assembled phonology (i.e., trained words in the assembled group) to a great extent reduced co-activations of the two reading routes. Consistent with these arguments, our results showed that the contrast between the trained words in the addressed group and those in the assembled group revealed much clearer results than the contrast between familiar words and pseudowords.

Results of our study made two significant contributions to the literature on the neural bases of reading. First, the artificial language training paradigm allowed us to better specify (and to resolve some related debates about) the neural substrates involved in the dual routes of phonological access during reading. In general, the two distinct neural pathways associated with addressed and assembled phonologies found in this study are consistent with the results of a previous meta-analysis on natural language materials [27], as well as previous findings of differential engagement of the ventral and dorsal neural pathways in lexical access and phonological processing [51]–[54]. Our results together with previous studies provide strong support for the dual-route models of reading, although they are not able to differentiate between localist (e.g., the dual-route cascaded model) [3] and connectionist models (e.g., the connectionist dual process model) [55], [56].

More importantly, our results may help resolve the continuing debate of the functional localization of the left temporoparietal cortex in assembled phonology. Although there is a general consensus about the involvement of the left temporoparietal cortex in grapheme-to-phoneme conversion [57]–[60], the exact location of temporoparietal activation varies greatly across existing studies (from the left posterior superior temporal gyrus (STG), AG, to SMG) [27]. Early lesion studies labeled the left AG as the center for grapheme-to-phoneme conversion [60]. However, the latest neuroimaging studies have suggested that the left AG and the left posterior STG (and adjacent SMG) are engaged in semantic and phonological processing, respectively [51], [60]–[62]. Consistent with this view, a recent meta-analysis on existing neuroimaging studies of dyslexia found consistent under-activation in the left STG and SMG for impaired readers [63]. A recent transcranial magnetic stimulation (TMS) study also found selective disruptions of phonological processing when TMS was applied over the SMG, but not when it was applied over AG [64]. Similarly, a recent neuroimaging study revealed a critical role of the left SMG as well as posterior MTG in orthography-phonology mapping after controlling for potential confounds such as task difficulty, word frequency, spelling-sound consistency, imageability, and length in letters [51]. In sum, the literature is mixed in terms of whether the STG is involved in assembled phonological access. When we used artificial language training to control for problems such as co-activations of both phonological routes and inherent variations in natural language materials, our results showed that activations in the left SMG, but not in the left posterior STG, were greater for assembled phonology than for addressed phonology. These results suggest that STG may not play a critical role in assembled phonology, and its activations in previous studies with natural languages might have been due to co-activations. It appears that the left SMG may be the main region involved in assembled phonology or orthography-phonology mapping.

Another refinement our results may provide to the dual-route model concerns the role of semantics. In the dual-neural-route model of reading, Jobard et al. [27] has proposed that regions in the ventral pathway are responsible for semantic access. There is ample evidence for the involvement of the ventral pathway in semantic access, but it is not clear whether the pathway is involved only in semantic-based access. By training our participants only in orthography-grapheme mapping (i.e., no semantics), we found that the ventral pathway (e.g., OFC, MTG) was involved in addressed phonology without the mediation of semantics. This finding poses an interesting question for future research: What are the overlapping and dissociated neural mechanisms between the semantic and nonsemantic routes in addressed phonology? This question can be addressed by comparing words trained with and without semantics. Results from such studies can help further refine the dual-route cascaded model (DRC) [3].

In addition to the refinements and specifications made to dual-route models of reading, the second contribution of our study is to provide direct experimental evidence for neural changes associated with the switch from assembled phonology to addressed phonology as a result of learning. Some dual-route models of reading [3], [29] have proposed that practice can shift participants' reading strategies from sub-lexical (i.e., assembled phonology) to more automatic lexical reading (i.e., addressed phonology). In particular, the phonologies of low-frequency regular words in alphabetic languages are mainly accessed through the assembled phonology route, whereas those of high-frequency words are mainly accessed through the addressed phonology route. In support of this view, our results found that, compared to untrained words, trained words elicited more activations in the addressed pathway and less activations in the assembled pathway. This result clearly shows the shift in neural pathway of reading as a result of increased familiarity with the new words. These results might also help to reconcile existing mixed findings in previous natural language studies that could have involved the co-activations of the two reading routes.

Three limitations of this study should be discussed. First, this study used a between-subject design to avoid the interference between addressed and assembled conditions. Although we matched the two groups of participants carefully, they might still have some differences that might potentially affect the results in this study. Future studies should consider a within-subject design to confirm the findings in this study. Second, trained and untrained words in the assembled group differed in task difficulty. We tried to remove the potential effect of task difficulty in the fMRI analysis by adding a covariate of RT. However, such a statistical control cannot eliminate all potential confounds. Finally, the present study successfully identified the neural pathways associated with the addressed and assembled phonologies by relying on the methodological merits of an artificial language training paradigm. However, the artificial language used in this study was different from natural languages in several important aspects. These differences might limit the generalization of our findings to natural languages to some extent. First, unlike natural languages, the artificial language used in this study only had a limited vocabulary size, which would eliminate some well-documented effects such as the neighborhood effect and regularity effect, and would impede the acquisition of the inherent structures of words such as the combination of letters (i.e., bigram, trigram). Second, although participants' accuracy in naming the artificial language words was generally high, their reading speed was still much slower than word reading in their native language. In other words, the two reading mechanisms found in this study were based on an early stage of word reading. There is evidence that reading networks are switched from the left temporoparietal cortex to the left occipitotemporal ventral areas with the improvement of reading skill [65], [66]. Therefore, the early and late stages of reading might differ in the engagement of the two neural routes. Future research on natural language development would help to clarify that question. Finally, as noted before, the DRC model [3] has proposed that addressed phonology can be accessed either directly (i.e., nonsemantic route) or mediated by semantics (i.e., semantic route) in natural language. Artificial language without semantics used in this study prevented us from exploring the neural substrate of the semantic route and examining the neural overlap and dissociation between the semantic and nonsemantic routes.

In sum, by using an artificial language training paradigm and thus overcoming the limitations of natural language materials, our study provides (1) a refined picture of neural substrates for addressed and assembled phonologies and (2) direct evidence of neural mechanisms involved in the strategy shift from assembled to addressed phonology during the process of learning to read.

## References

[1] Marshall JC, Newcombe F (1973) Patterns of paralexia: A psycholinguistic approach. J Psycholinguist Res 2: 175–199. 4795473 10.1007/BF01067101

[2] Shallice T, Warrington EK (1975) Word recognition in a phonemic dyslexic patient. Q J Exp Psychol 27: 187–199. 1187994 10.1080/14640747508400479

[3] Coltheart M, Rastle K, Perry C, Langdon R, Ziegler J (2001) DRC: A dual route cascaded model of visual word recognition and reading aloud. Psychol Rev 108: 204–256. 11212628 10.1037/0033-295x.108.1.204

[4] Coltheart M, Curtis B, Atkins P, Haller M (1993) Models of reading aloud: Dual-route and parallel-distributed-processing approaches. Psychol Rev 100: 589–608.

[5] Brunswick N, McCrory E, Price CJ, Frith CD, Frith U (1999) Explicit and implicit processing of words and pseudowords by adult developmental dyslexics: A search for Wernicke's Wortschatz? Brain 122: 1901–1917. 10506092 10.1093/brain/122.10.1901

[6] Fiebach CJ, Friederici AD, Miller K, Cramon DYv (2002) fMRI Evidence for Dual Routes to the Mental Lexicon in Visual Word Recognition. J Cogn Neurosci 14: 11–23. 11798383 10.1162/089892902317205285

[7] Fiez JA, Balota DA, Raichle ME, Petersen SE (1999) Effects of Lexicality, Frequency, and Spelling-to-Sound Consistency on the Functional Anatomy of Reading. Neuron 24: 205–218. 10677038 10.1016/s0896-6273(00)80833-8

[8] Hagoort P, Indefrey P, Brown C, Herzog H, Steinmetz H, et al (1999) The Neural Circuitry Involved in the Reading of German Words and Pseudowords: A PET Study. J Cogn Neurosci 11: 383–398. 10471847 10.1162/089892999563490

[9] Herbster AN, Mintun MA, Nebes RD, Becker JT (1997) Regional cerebral blood flow during word and nonword reading. Hum Brain Mapp 5: 84–92. 10096413 10.1002/(sici)1097-0193(1997)5:2<84::aid-hbm2>3.0.co;2-i

[10] Mechelli A, Crinion JT, Long S, Friston KJ, Ralph MAL, et al (2005) Dissociating Reading Processes on the Basis of Neuronal Interactions. J Cogn Neurosci 17: 1753–1765. 16269111 10.1162/089892905774589190

[11] Mechelli A, Gorno-Tempini ML, Price CJ (2003) Neuroimaging Studies of Word and Pseudoword Reading: Consistencies, Inconsistencies, and Limitations. J Cogn Neurosci 15: 260–271. 12676063 10.1162/089892903321208196

[12] Nosarti C, Mechelli A, Green DW, Price CJ (2010) The Impact of Second Language Learning on Semantic and Nonsemantic First Language Reading. Cereb Cortex 20: 315–327. 19478033 10.1093/cercor/bhp101 PMC2803733

[13] Simos PG, Breier JI, Fletcher JM, Foorman BR, Castillo EM, et al (2002) Brain Mechanisms for Reading Words and Pseudowords: an Integrated Approach. Cereb Cortex 12: 297–305. 11839603 10.1093/cercor/12.3.297

[14] Levy J, Pernet C, Treserras S, Boulanouar K, Aubry F, et al (2009) Testing for the Dual-Route Cascade Reading Model in the Brain: An fMRI Effective Connectivity Account of an Efficient Reading Style. PLoS ONE 4: e6675. 19688099 10.1371/journal.pone.0006675 PMC2724737

[15] Cattinelli I, Borghese NA, Gallucci M, Paulesu E (2013) Reading the reading brain: A new meta-analysis of functional imaging data on reading. J Neurolinguistics 26: 214–238.

[16] Nakamura K, Dehaene S, Jobert A, Bihan DL, Kouider S (2005) Subliminal Convergence of Kanji and Kana Words: Further Evidence for Functional Parcellation of the Posterior Temporal Cortex in Visual Word Perception. J Cogn Neurosci 17: 954–968. 15969912 10.1162/0898929054021166

[17] Sakurai Y, Momose T, Iwata M, Sudo Y, Ohtomo K, et al (2000) Different cortical activity in reading of Kanji words, Kana words and Kana nonwords. Cogn Brain Res 9: 111–115. 10.1016/s0926-6410(99)00052-x 10666563

[18] Thuy DH, Matsuo K, Nakamura K, Toma K, Oga T, et al (2004) Implicit and explicit processing of kanji and kana words and non-words studied with fMRI. Neuroimage 23: 878–889. 15528088 10.1016/j.neuroimage.2004.07.059

[19] Fu S, Chen Y, Smith S, Iversen S, Matthews PM (2002) Effects of Word Form on Brain Processing of Written Chinese. Neuroimage 17: 1538–1548. 12414292 10.1006/nimg.2002.1155

[20] Chen Y, Fu S, Iversen SD, Smith SM, Matthews PM (2002) Testing for Dual Brain Processing Routes in Reading: A Direct Contrast of Chinese Character and Pinyin Reading Using fMRI. J Cogn Neurosci 14: 1088–1098. 12419131 10.1162/089892902320474535

[21] Paulesu E, McCrory E, Fazio F, Menoncello L, Brunswick N, et al (2000) A cultural effect on brain function. Nat Neurosci 3: 91–96. 10607401 10.1038/71163

[22] Price CJ, Wise RJS, Frackowiak RSJ (1996) Demonstrating the Implicit Processing of Visually Presented Words and Pseudowords. Cereb Cortex 6: 62–70. 8670639 10.1093/cercor/6.1.62

[23] Xu B, Grafman J, Gaillard WD, Ishii K, Vega-Bermudez F, et al (2001) Conjoint and Extended Neural Networks for the Computation of Speech Codes: The Neural Basis of Selective Impairment in Reading Words and Pseudowords. Cereb Cortex 11: 267–277. 11230098 10.1093/cercor/11.3.267

[24] Carreiras M, Mechelli A, Estevez A, Price CJ (2007) Brain Activation for Lexical Decision and Reading Aloud: Two Sides of the Same Coin? J Cogn Neurosci 19: 433–444. 17335392 10.1162/jocn.2007.19.3.433

[25] Heim S, Alter K, Ischebeck AK, Amunts K, Eickhoff SB, et al (2005) The role of the left Brodmann's areas 44 and 45 in reading words and pseudowords. Cogn Brain Res 25: 982–993. 10.1016/j.cogbrainres.2005.09.022 16310346

[26] Joubert S, Beauregard M, Walter N, Bourgouin P, Beaudoin G, et al (2004) Neural correlates of lexical and sublexical processes in reading. Brain Lang 89: 9–20. 15010232 10.1016/S0093-934X(03)00403-6

[27] Jobard G, Crivello F, Tzourio-Mazoyer N (2003) Evaluation of the dual route theory of reading: a metanalysis of 35 neuroimaging studies. Neuroimage 20: 693–712. 14568445 10.1016/S1053-8119(03)00343-4

[28] Tagamets MA, Novick JM, Chalmers ML, Friedman RB (2000) A Parametric Approach to Orthographic Processing in the Brain: An fMRI Study. J Cogn Neurosci 12: 281–297. 10771412 10.1162/089892900562101

[29] Binder JR, Medler DA, Desai R, Conant LL, Liebenthal E (2005) Some neurophysiological constraints on models of word naming. Neuroimage 27: 677–693. 15921937 10.1016/j.neuroimage.2005.04.029

[30] Paap KR, Noel RW (1991) Dual route models of print to sound: Still a good horse race. Psychol Res 53: 13–24.

[31] Seidenberg MS, Waters GS, Barnes MA, Tanenhaus MK (1984) When does irregular spelling or pronunciation influence word recognition? J Verbal Learning Verbal Behav 23: 383–404.

[32] Taraban R, McClelland JL (1987) Conspiracy effects in word recognition. J Mem Lang 26: 608–631.

[33] Chen C, Xue G, Mei L, Chen C, Dong Q (2009) Cultural neurolinguistics. In: Y. C Joan, editor editors. Progress in Brain Research.Elsevier. pp. 159–171. 10.1016/S0079-6123(09)17811-1 PMC2821076 19874968

[34] Xue G, Chen C, Jin Z, Dong Q (2006) Language experience shapes fusiform activation when processing a logographic artificial language: An fMRI training study. Neuroimage 31: 1315–1326. 16644241 10.1016/j.neuroimage.2005.11.055

[35] Maloney E, Risko EF, O'Malley S, Besner D (2009) Tracking the transition from sublexical to lexical processing: On the creation of orthographic and phonological lexical representations. Q J Exp Psychol 62: 858–867. 10.1080/17470210802578385 19107643

[36] Raven JC (1990) Advanced Progressive Matrices: Sets I, II. Oxford: Oxford University Press.

[37] Woodcock R (1987) Woodcock Reading Mastery Tests–Revised. Circle Pines, MN: American Guidance Service.

[38] Torgesen J, Wagner R, Rashotte C (1999) Test of word reading efficiency. Austin, TX: Pro-Ed.

[39] Snyder PJ, Harris LJ (1993) Handedness, sex, and familial sinistrality effects on spatial tasks. Cortex 29: 115–134. 8472549 10.1016/s0010-9452(13)80216-x

[40] Wilson M (1988) MRC psycholinguistic database: Machine-usable dictionary, version 2.00. Beh Res Meth 20: 6–10.

[41] Rastle K, Harrington J, Coltheart M (2002) 358,534 nonwords: The ARC Nonword Database Q J Exp Psychol A. 55: 1339–1362. 10.1080/02724980244000099 12420998

[42] Cohen L, Lehericy S, Chochon F, Lemer C, Rivaud S, et al (2002) Language-specific tuning of visual cortex? Functional properties of the Visual Word Form Area. Brain 125: 1054–1069. 11960895 10.1093/brain/awf094

[43] Chen C, Xue G, Dong Q, Jin Z, Li T, et al (2007) Sex determines the neurofunctional predictors of visual word learning. Neuropsychologia 45: 741–747. 16999980 10.1016/j.neuropsychologia.2006.08.018

[44] Dale AM (1999) Optimal experimental design for event-related fMRI. Hum Brain Mapp 8: 109–114. 10524601 10.1002/(SICI)1097-0193(1999)8:2/3<109::AID-HBM7>3.0.CO;2-W PMC6873302

[45] Jenkinson M, Smith S (2001) A global optimisation method for robust affine registration of brain images. Med Image Anal 5: 143–156. 11516708 10.1016/s1361-8415(01)00036-6

[46] Tohka J, Foerde K, Aron AR, Tom SM, Toga AW, et al (2008) Automatic independent component labeling for artifact removal in fMRI. Neuroimage 39: 1227–1245. 18042495 10.1016/j.neuroimage.2007.10.013 PMC2374836

[47] Beckmann CF, Jenkinson M, Smith SM (2003) General multilevel linear modeling for group analysis in FMRI. Neuroimage 20: 1052–1063. 14568475 10.1016/S1053-8119(03)00435-X

[48] Woolrich MW (2008) Robust group analysis using outlier inference. Neuroimage 41: 286–301. 18407525 10.1016/j.neuroimage.2008.02.042

[49] Woolrich MW, Behrens TEJ, Beckmann CF, Jenkinson M, Smith SM (2004) Multilevel linear modelling for FMRI group analysis using Bayesian inference. Neuroimage 21: 1732–1747. 15050594 10.1016/j.neuroimage.2003.12.023

[50] Mumford J (2007) A Guide to Calculating Percent Change with Featquery. Unpublished Tech Report available at http://mumfordboluclaedu/perchange_guidepdf.

[51] Graves WW, Desai R, Humphries C, Seidenberg MS, Binder JR (2010) Neural Systems for Reading Aloud: A Multiparametric Approach. Cereb Cortex 20: 1799–1815. 19920057 10.1093/cercor/bhp245 PMC2901017

[52] Poldrack RA, Wagner AD, Prull MW, Desmond JE, Glover GH, et al (1999) Functional Specialization for Semantic and Phonological Processing in the Left Inferior Prefrontal Cortex. Neuroimage 10: 15–35. 10385578 10.1006/nimg.1999.0441

[53] McDermott KB, Petersen SE, Watson JM, Ojemann JG (2003) A procedure for identifying regions preferentially activated by attention to semantic and phonological relations using functional magnetic resonance imaging. Neuropsychologia 41: 293–303. 12457755 10.1016/s0028-3932(02)00162-8

[54] Burton H, Diamond JB, McDermott KB (2003) Dissociating Cortical Regions Activated by Semantic and Phonological Tasks: A fMRI Study in Blind and Sighted People. J Neurophysiol 90: 1965–1982. 12789013 10.1152/jn.00279.2003 PMC3705560

[55] Perry C, Ziegler JC, Zorzi M (2007) Nested Incremental Modeling in the Development of Computational Theories: The CDP+ Model of Reading Aloud. Psychol Rev 114: 273–315. 17500628 10.1037/0033-295X.114.2.273

[56] Zorzi M, Houghton G, Butterworth B (1998) Two routes or one in reading aloud? A connectionist ‘dual-process’ model. J Exp Psychol Hum Percept Perform 24: 1131–1161.

[57] Bolger DJ, Perfetti CA, Schneider W (2005) Cross-cultural effect on the brain revisited: universal structures plus writing system variation. Hum Brain Mapp 25: 92–104. 15846818 10.1002/hbm.20124 PMC6871743

[58] Tan LH, Laird AR, Li K, Fox PT (2005) Neuroanatomical correlates of phonological processing of Chinese characters and alphabetic words: a meta-analysis. Hum Brain Mapp 25: 83–91. 15846817 10.1002/hbm.20134 PMC6871734

[59] Price CJ (1998) The functional anatomy of word comprehension and production. Trends Cogn Sci 2: 281–288. 21227210 10.1016/s1364-6613(98)01201-7

[60] Price CJ (2000) The anatomy of language: contributions from functional neuroimaging. J Anat 197 Pt 3: 335–359. 10.1046/j.1469-7580.2000.19730335.x PMC1468137 11117622

[61] Lee H, Devlin JT, Shakeshaft C, Stewart LH, Brennan A, et al (2007) Anatomical Traces of Vocabulary Acquisition in the Adolescent Brain. J Neurosci 27: 1184–1189. 17267574 10.1523/JNEUROSCI.4442-06.2007 PMC6673201

[62] Seghier ML, Fagan E, Price CJ (2010) Functional Subdivisions in the Left Angular Gyrus Where the Semantic System Meets and Diverges from the Default Network. J Neurosci 30: 16809–16817. 21159952 10.1523/JNEUROSCI.3377-10.2010 PMC3105816

[63] Richlan F, Kronbichler M, Wimmer H (2009) Functional abnormalities in the dyslexic brain: A quantitative meta-analysis of neuroimaging studies. Hum Brain Mapp 30: 3299–3308. 19288465 10.1002/hbm.20752 PMC2989182

[64] Hartwigsen G, Baumgaertner A, Price CJ, Koehnke M, Ulmer S, et al (2010) Phonological decisions require both the left and right supramarginal gyri. Proc Natl Acad Sci U S A 107: 16494–16499. 20807747 10.1073/pnas.1008121107 PMC2944751

[65] Pugh KR, Mencl WE, Jenner AR, Katz L, Frost SJ, et al (2001) Neurobiological studies of reading and reading disability. J Commun Disord 34: 479–492. 11725860 10.1016/s0021-9924(01)00060-0

[66] Church JA, Coalson RS, Lugar HM, Petersen SE, Schlaggar BL (2008) A Developmental fMRI Study of Reading and Repetition Reveals Changes in Phonological and Visual Mechanisms Over Age. Cereb Cortex 18: 2054–2065. 18245043 10.1093/cercor/bhm228 PMC2517103

[67] Van Essen DC (2005) A Population-Average, Landmark- and Surface-based (PALS) atlas of human cerebral cortex. Neuroimage 28: 635–662. 16172003 10.1016/j.neuroimage.2005.06.058

[68] Van Essen DC (2002) Windows on the brain: the emerging role of atlases and databases in neuroscience. Curr Opin Neurobiol 12: 574–579. 12367638 10.1016/s0959-4388(02)00361-6

[69] Van Essen DC, Drury HA, Dickson J, Harwell J, Hanlon D, et al (2001) An Integrated Software Suite for Surface-based Analyses of Cerebral Cortex. J Am Med Inform Assoc 8: 443–459. 11522765 10.1136/jamia.2001.0080443 PMC131042

